# Editorial: Vascularization for Regenerative Medicine

**DOI:** 10.3389/fbioe.2018.00175

**Published:** 2018-11-21

**Authors:** Andrea Banfi, Wolfgang Holnthoner, Mikaël M. Martino, Seppo Ylä-Herttuala

**Affiliations:** ^1^Department of Biomedicine, University Hospital, University of Basel, Basel, Switzerland; ^2^AUVA Research Centre, Ludwig Boltzmann Institute for Experimental and Clinical Traumatology, Vienna, Austria; ^3^European Molecular Biology Laboratory Australia, Australian Regenerative Medicine Institute, Monash University, Melbourne, VIC, Australia; ^4^A.I. Virtanen Institute for Molecular Sciences, Univeristy of Eastern Finland, Kuopio, Finland

**Keywords:** angiogenesis, lymphangiogeneis, vascularization, tissue engineeering, regenerative medicine

The generation of functional vascular networks is crucial for regenerative medicine, especially for the survival of engineered tissue grafts on one hand and blood flow restoration to ischemic tissues on the other.

Tissue engineering aims at developing biological substitutes to replace traumatic, neoplastic, or degenerative tissue loss, by the *in vitro* culture of appropriate progenitors with suitable material scaffolds. However, upon implantation *in vivo*, a major challenge for clinically relevant large-size grafts is the maintenance of cell viability in the core of the scaffold, which critically depends on the rapid invasion of the construct by the host blood vessels. In fact, diffusion from the surrounding blood vascular bed only reaches the outer shell of the graft, resulting in cell death in the central core of the implants and limiting tissue ingrowth to the outer 1–2 mm (Grosso et al., 2017). Furthermore, the presence of a functional vasculature also allows the recruitment of highly specialized cells, like circulating tissue progenitors or reparative myeloid cells, which can contribute to tissue regeneration and remodeling (Lo Sicco et al., 2015). The regulation of vascular growth and tissue regeneration by immune cells is also an emerging area of significant interest (Lo Sicco and Tasso, 2017). Finally, removal of interstitial fluid, comprising extravasated cells, proteins and lipids, through uptake by lymphatic capillaries and return to the blood stream by larger lymphatic vessels represents one of the challenges in vascular regeneration (Schaupper et al., 2016). Therefore, rapid vascularization within days of *in vivo* implantation is of vital importance for survival and function of tissue-engineered grafts of clinically relevant size and one of the major limiting factors toward their implementation for patient treatment.

On the other hand, ischemic conditions, due for example to atherosclerotic artery occlusion, still cause significant morbidity and mortality, which are not solved by current medical and surgical treatments (Benjamin et al., 2018). Hence there is an urgent need for new treatment strategies: therapeutic angiogenesis aims at stimulating the growth of new microvascular networks by local delivery of angiogenic factors and the subsequent functional remodeling of new collateral arteries, i.e. arteriogenesis (Annex, 2013).

Angiogenesis and lymphangiogenesis are complex and highly regulated processes (Potente et al., 2011). The design of rational therapeutic approaches stands to greatly benefit from a thorough understanding of the cellular and molecular mechanisms underlying the physiological growth and remodeling of vascular structures under therapeutic conditions.

This Frontiers Research Topic brings together contributions investigating different facets of promoting vascularization in tissue reconstruction and repair, spanning from basic mechanisms of blood and lymphatic vessel biology, to the study and quantification of vascularization dynamics *in vivo* under therapeutic conditions, to the translational aspects and challenges of achieving efficient vascularization of tissue-engineered grafts or even whole organs.

The importance of angiogenesis and lymphangiogenesis in regenerative medicine is highlighted by four articles, which describe several transcription factors, growth factors, and microRNAs important for stem cell and progenitor cell differentiation to mature endothelial cells, capillary sprouting and formation of large arteries and lymphatic vessels. Sturtzel et al. describe the importance of forkhead box transcription factor (FOXF1) in endothelial colony-forming cells, where it was found to stimulate vascular sprouting by inducing Notch2 receptors and augmenting expression of VEGF Receptor-2 and EphrinB2. Results were verified with knockdown experiments and in a zebrafish model. Along the same lines Belt et al. describe dynamics of gene expression during endothelial cell differentiation from human iPS cells. They provide the first systematic characterization and comparative analysis of seven different small molecule-mediated differentiation protocols to induce optimal endothelial cell phenotype. Key points appear to be early inhibition of Rho-associated kinase, activation of cyclic AMP signaling and inhibition of TGF-β signaling.

Improved tissue perfusion is not possible without generation of large arteries since capillaries alone cannot provide enough perfusion for large tissue masses. Heuslein et al. show that anti-miRNA-146a improved ischemic peripheral muscle perfusion and collateral artery diameters even though it did not have any significant effects on angiogenesis. Thus, different mechanisms appear to regulate large blood vessel formation and angiogenesis. This should be taken into account in therapeutic approaches. It has recently been recognized that equally important to stimulation of blood vessel growth is the adequate function of lymphatic vessels in ischemic tissues. Rauniyar et al. review the important role of VEGF-C in regulating the growth of lymphatic vessels. They show that VEGF-C biosynthesis has several steps and protolytic processing significantly affects VEGF-C biodistribution, receptor activation and growth of lymphatic vessels. Recognizing these properties will be essential for the utilization of VEGF-C for regenerative purposes *in vivo*.

Co-cultures of endothelial cells and supporting cells are widely used to generate microvasculature and pre-vascularized engineered grafts. Knezevic et al. show that blood vascular endothelial cells form stable and sustainable networks by using a co-culture model with adipose-derived stromal cells in fibrin matrix. Not only blood vessel networks, but also lymphatic networks are required for tissue constructs and the team shows that it can be achieved via VEGF-C. Landau et al. show migration of endothelial cells in the depths of the scaffold, resulting in microvasculature surrounded by supporting cells with different location-dependent phenotypes. The assessment of angiogenesis *in vivo* can still be challenging. Slezak et al. present a novel low-cost rat model to study angiogenesis, enabling spatial assessment of vascularization by a simple implantation procedure. Mesenchymal stem cells provide beneficial paracrine factors that improve cardiac function and regeneration, but their therapeutic potential depends on the method of delivery. Zlabinger et al. demonstrate that intracoronary delivery leads to diminished blood flow and impaired cell homing compared to intramyocardial injection through elevated MMP-2 and decreased expression of the homing factor CXCR4.

Finally, Pellegata et al. review the numerous approaches aiming at regenerating the full vascular tree of decellularized tissues for whole-organ engineering, while Carrabba and Madeddu review scaffold-based and cell-based approaches that have been developed to create tissue-engineered vascular grafts in the last decades. Notably, the review highlights the attempts made to translate the strategies from bench to bedside.

## Author contributions

All authors listed wrote the manuscript and approved it for publication.

### Conflict of interest statement

The authors declare that the research was conducted in the absence of any commercial or financial relationships that could be construed as a potential conflict of interest.
